# Ultrasound-Guided Occipital Nerve Block for Treatment of Atypical Occipital Neuralgia

**DOI:** 10.7759/cureus.18584

**Published:** 2021-10-07

**Authors:** Colby Skinner, Sanjeev Kumar

**Affiliations:** 1 Anesthesiology, University of Florida College of Medicine, Gainesville, USA

**Keywords:** facial pain, occipital neuralgia, headache, nerve block, local anesthetics

## Abstract

Occipital neuralgia can occur secondary to injury to the occipital nerves or the C2 or C3 nerve roots. Symptoms of occipital neuralgia can include sudden and debilitating craniofacial pain, otalgia, neck pain, shoulder pain, vision changes, and tinnitus. We describe how an ultrasound-guided greater occipital nerve block substantially reduced symptoms associated with a middle-aged woman’s atypical presentation of occipital neuralgia, which was refractory to oral medications and other procedural interventions.

## Introduction

Patients with occipital neuralgia often experience a wide variety of symptoms, including shooting facial, neck, or shoulder pain, tinnitus, vision changes, and otalgia [1]. These symptoms can be treated with a variety of treatment modalities including oral medications and interventional management. We explore the capabilities of a greater occipital nerve block to provide patients with relief from symptoms associated with occipital neuralgia. This case demonstrates that greater occipital nerve block may be an efficacious and low-risk long-term treatment for patients presenting with occipital neuralgia. This simple nerve block can be an option when alternative treatments do not provide relief.

Written informed consent and Health Insurance Portability and Accountability Act authorization were obtained from the patient to report this case. This article adheres to the applicable Enhancing the QUAlity and Transparency Of health Research (EQUATOR) guideline.

## Case presentation

The patient is a healthy 54-year-old woman who presented to our academic interventional pain clinic for evaluation of occipital neuralgia and atypical facial pain. Her symptoms began while scuba diving about a year ago. She started having non-radiating bilateral temporal and occipital headache, ear pain, tinnitus, and neck, jaw, and shoulder pain along with stiffness. She also noticed exertional dizziness and persistent left-eye blurry vision for which she consulted with an ophthalmologist and neurologist and no reasonable explanations or treatments were offered for those symptoms. She had bilateral clinical features but the left-sided symptoms were more severe than that of the right side. The patient also experienced thoracic paraspinal muscle spasms and trigger points were elicited in the same region upon palpation. She described her pain as heavy and pressure-like, as well as tingling with a 9 out of 10 numerical rating of pain severity at the worst and 4 out of 10 on average. Her current and past medication regimen included ibuprofen, methocarbamol, cyclobenzaprine, acetaminophen, benzodiazepines, gabapentin, and cannabidiol oil, which were only minimally effective in reducing or managing her pain. She had previously undertaken interventional management including a sphenopalatine block, trigger point injections, and acupuncture without benefit. The patient had also previously received C3, C4, and C5 medial branch blocks without therapeutic effect. The patient underwent brain MRI, cervical spine MRI, and electromyography, all of which were within normal limits.

**Figure 1:**
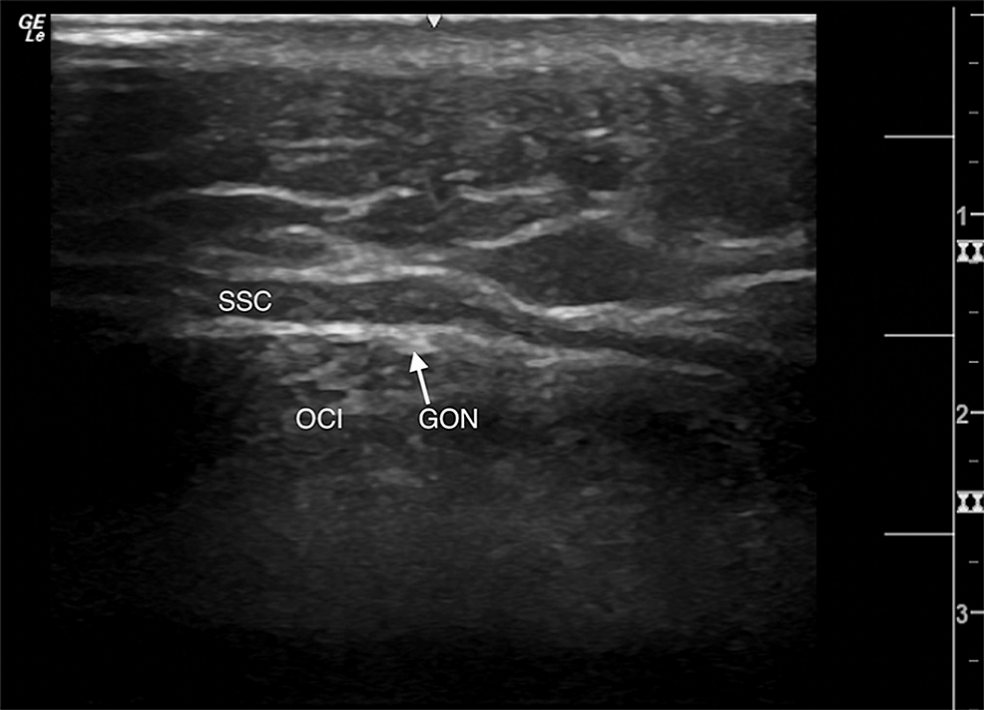
Left-sided greater occipital nerve (GON) seen with high-frequency linear ultrasound probe SSC: semispinalis capitis; OCI: obliquus capitis inferior.

The patient’s diagnosis of occipital neuralgia was consistent with the diagnostic criteria in the International Classification of Headache Disorders, 3rd edition [2]. After discussing the risks (bleeding, infection, neuritis, worsening of pain, accidental entry to the spinal canal, and related complications) and benefits (relief in symptoms) with the patient, an occipital nerve block was performed using 1 mL of methylprednisolone (40 mg/mL) and 2 mL of 0.5% bupivacaine. The transverse process of the C2 vertebrae was found with ultrasound and the high-frequency linear probe was used to find the greater occipital nerve between the muscle planes of the obliquus capitis inferior and semispinalis capitis (Figure 1).

Before the procedure, the patient rated her pain as a 9 out of 10. Within a few minutes after the procedure, her pain was reduced to a 2 out of 10, representing a 77.8% reduction in her self-assessed numerical pain score. The patient also reported immediate improvement in her visual acuity, perceived as "clearing of vision" in her left eye, as well as relief of her left ear pain and tinnitus. The patient’s scalp, facial and ear pain relief, as well as her tinnitus and vision improvement, remain sustained two years after the procedure.

## Discussion

Occipital neuralgia is a condition in which inflammation or injury of the occipital nerves [3] or the C2 or C3 nerve roots [4] causes shock-like, stabbing, or shooting facial, neck, or shoulder pain, tinnitus, vision changes, and otalgia [5]. The onset is spontaneous but it can subsequently be paroxysmal or provoked by palpation, neck movements, or temperature changes [2]. Occipital neuralgia is rare, with some literature finding a prevalence of 1.2%, and women representing 80% of cases [6]. Pain can be unilateral or bilateral, starting at the skull base and spreading superiorly. The range of motion of the cervical spine may be limited by pain. Occipital neuralgia can be associated with loss of sensation in the affected area.

The greater occipital nerve block is both diagnostic of and therapeutic for occipital neuralgia. Greater occipital nerve blocks involve an injection of a local anesthetic agent, often mixed with steroids, to the perineurial space of the greater occipital nerve preferably under ultrasound guidance. Relief can start within minutes but the duration of symptoms improvement after a greater occipital nerve block is variable and can often last for months [7]. Greater occipital nerve blocks can be repeated as needed for pain recurrence every three months [8]. The greater occipital nerve block with ultrasound guidance involves identification of the bifid spinous process and lamina of the C2 vertebra and then the greater occipital nerve between the muscle planes of the obliquus capitis inferior and semispinalis capitis.

Our approach was aimed at treating the patient’s occipital neuralgia to provide relief from her symptoms. Our patient’s severe symptoms were disruptive to her ability to perform tasks at home and work, her relationships with others, her sleep, and her enjoyment of life. Accordingly, before presenting at our clinic, the patient had been unsuccessfully treated with numerous therapeutic approaches to attempt to alleviate her symptoms. Interestingly, a single ultrasound-guided nerve block resolved our patient’s occipital neuralgia symptoms, including vision disturbance and tinnitus, and almost all of her pain. The benefits of the single injection are still sustained at two years. Although we can't explain the rationale of sustained relief with just a single injection, one possible explanation could be breaking the "vicious cycle" of pain and myriad symptoms of occipital neuralgia by a well-conducted nerve block. 

## Conclusions

As in our patient’s case, the ultrasound-guided occipital nerve block is a low-risk therapeutic intervention for occipital neuralgia and atypical facial pain. The risks mentioned earlier: bleeding, infection, neuritis, worsening of pain, accidental entry to the spinal canal and related complications can be almost entirely eliminated with good visualization of the needle tip and proper identification of vascular and neural structures on ultrasound. It can be a valuable option for treating the wide-ranging and myriad symptoms that patients can experience due to occipital neuralgia, especially when other options for treatment have been unsuccessful. This case report shows that greater occipital nerve block may result in years-long therapeutic improvement in quality of life for patients with occipital neuralgia.
